# Effects of Simulated Service Environments on the Microstructure and Interfacial Properties of Ceramic Fiber-Reinforced Al-Matrix Composites

**DOI:** 10.3390/ma19101999

**Published:** 2026-05-12

**Authors:** Desheng Chu, Yanhan Wang, Fangrong Zhou, Ronghai Liu, Longchang Zhu, Qingjun Peng

**Affiliations:** 1 Electric Power Research Institute of Yunnan Power Grid Co., Ltd., Kunming 650500, China; 2 School of Electrical Engineering, Chongqing University, Chongqing 400044, China

**Keywords:** SiC_f_/Al composite, thermal exposure, thermal cycling, interfacial shear strength, push-out test, interfacial reaction layer, thermal residual stress

## Abstract

SiC fiber-reinforced aluminum matrix (SiC_f_/Al) composites have the potential to replace titanium alloys for fan/compressor blades due to their low density and favorable high-temperature performance. In this study, thermal exposure and thermal cycling tests were conducted to simulate service environments and to clarify their effects on the microstructure and interfacial properties of a SiC_f_/AlFe5Si2 composite. Thermal exposure was performed at 260–450 °C for 20–100 h, and thermal cycling was carried out between 300 or 350 °C (1 h dwell) and room temperature for 20–100 cycles. Interfacial shear strength was evaluated by push-out tests, while microstructural evolution was examined using SEM, TEM/EDS, and XRD. Three-dimensional finite element simulations were used to assess mismatch-driven residual-stress distributions during the cooling stage after thermal excursion. The results showed that interfacial shear strength decreased with increasing exposure temperature/time and degraded more severely under thermal cycling than under isothermal exposure at the same temperature. A rapid loss of interfacial strength occurred above ~400 °C, associated with significant interfacial-layer thickening and the formation of brittle Al_x_SiO_y_ phases. The interfacial reaction layer followed parabolic growth kinetics, yielding a preliminary apparent activation energy of Q ≈ 150 kJ/mol estimated from two isothermal temperatures. The simulations indicated large opposing stresses between the matrix and the carbon-rich layer, supporting a mechanical driving force for interfacial debonding; however, heating/dwell time-dependent effects were not explicitly modeled and are discussed as limitations. These findings provide quantitative guidance for defining service-temperature limits and improving interfacial thermal stability in SiC_f_/AlFe5Si2 composites.

## 1. Introduction

SiC_f_/Al composites exhibit high specific strength and modulus, together with better heat resistance than conventional aluminum alloys, which makes them promising for aerospace applications. The operating temperature of the low-temperature section of aero-engine fans/compressors typically reaches 200–300 °C, where titanium alloys are currently the dominant materials for fan/compressor blades. Given a density of 3.1 g/cm^3^ for SiC_f_/Al compared with 4.5 g/cm^3^ for titanium alloys, replacing titanium alloys with SiC_f_/Al is expected to achieve an approximately 30% weight reduction, which would benefit the thrust-to-weight ratio of engines.

Recent studies have focused on the thermal stability of SiC_f_/Al interfaces and the effects of thermal cycling on composite performance, which are directly relevant to understanding the service behavior of SiC_f_/Al composites. Wang et al. [1] demonstrated that molecular thermal linkers can enhance interfacial thermal conductivity and stability in liquid metal composites, providing insights for improving interfacial bonding in SiC/Al systems. Liu et al. [2] specifically investigated the interfacial thermal stability of SiC fiber reinforced aluminum matrix composites with carbon coatings, finding that appropriate interfacial modifications can significantly improve high-temperature performance. Zhang et al. [3] provided valuable insights into the microstructure and mechanical properties of composites with engineered interphases, which are crucial for understanding the interfacial reaction layer evolution observed in SiC_f_/Al composites. Furthermore, Zhong et al. [4] comprehensively reviewed recent advances in processing and characterization of metal matrix composites for high-temperature applications, highlighting the importance of thermal stability and interfacial engineering for aerospace materials.

Microstructural and property stability under service conditions strongly affects the reliability of fan/compressor blade materials. Therefore, thermal exposure and thermal cycling tests were adopted to simulate service environments and to investigate the evolution of microstructure and interfacial properties in SiC_f_/Al composites. Chen et al. studied a 0.6 vol.% SiC_f_/7075Al composite after aging in an oil bath at 120 °C and reported that the interfacial shear strength increased with aging time up to 25 h, consistent with the strengthening trend of the Al matrix [5]. In contrast, Jiang et al. found that the interfacial shear strength of a SiC_f_/LD-2Al composite decreased from 19.78 MPa to 7.84 MPa after heat treatment [6]. Notably, the heat-treatment temperatures in these studies were relatively low; for 7075 and LD-2 alloys, over-aging above 150 °C usually causes a reduction in alloy strength. Only a few studies have reported on the microstructure and interfacial characteristics of SiC_f_/Al composites at service temperatures above 300 °C.

This work employed thermal exposure and thermal cycling tests to study the microstructural evolution and interfacial characteristics of the SiC_f_/AlFe5Si2 composite. Thermal exposure was conducted at 260, 300, 350, 400, and 450 °C for 20, 50, and 100 h. Thermal cycling was performed by heating to 300 or 350 °C, holding for 1 h, and cooling to room temperature (~30 °C) as one cycle, for 20, 50, and 100 cycles. Microstructure and properties before and after testing were characterized. Three-dimensional finite element method (3D FEM) simulations were further carried out to evaluate thermal residual-stress distributions during cooling from peak temperatures relevant to the thermal exposure/cycling conditions, aiming to provide a mechanistic complement to the experimental observations of microstructure and interfacial performance. Compared with our previous study on SiC_f_/AlFe5Si2 (Ref. [7]), which primarily examined interfacial microstructure and interfacial shear strength after isothermal thermal exposure, the present work extends the scope in four aspects. First, it introduces a systematic comparison between isothermal exposure and thermal cycling, enabling direct assessment of cumulative cycling effects. Second, it provides an integrated mechanism framework that links matrix softening, reaction-layer thickening, brittle Al_x_SiO_y_ formation, and interphase damage. Third, it incorporates a 3D RVE-based FEM analysis to interpret mismatch-driven residual-stress contrast as a mechanical contributor to debonding tendency. Finally, it proposes condition-specific engineering guidance for exposure versus cycling, together with explicit applicability and limitation statements.

## 2. Experimental and Numerical Methods

### 2.1. Material and Specimen Preparation

Since SiC_f_/Al composite materials are primarily applied as high-temperature resistant structural materials, to investigate the interfacial properties and microstructural evolution of SiC_f_/Al composite materials under service operating environments, this study simulates the service environment through thermal exposure and thermal cycling. Compared with other common aluminum alloy matrices such as 6061Al and 2024Al, AlFe5Si2 belongs to heat-resistant aluminum alloys and exhibits the best high-temperature performance. Therefore, the thermal exposure and thermal cycling experiments mainly focus on SiC_f_/AlFe5Si2 composite materials.

Using electrical discharge wire cutting, a total of 36 thin slices (thickness ~0.5 mm) were cut from the cross-section perpendicular to the fiber axial direction of the as-fabricated 35 vol.% SiC_f_/AlFe5Si2 composite material. These thin slices were divided into 18 groups, with each group containing two slices: one for push-out testing and another for microstructural observation. Except for one group serving as a comparison without thermal exposure testing, the other groups underwent thermal exposure and thermal cycling tests, respectively. The thermal exposure test process was: thermal exposure at 260, 300, 350, 400, and 450 °C for 20, 50, and 100 h in an air heating furnace, followed by furnace cooling to room temperature. The thermal cycling test process was: heating at 300 °C and 350 °C for 1 h, respectively, in an air heating furnace, then removing for air cooling to room temperature, and then placing back into the furnace, repeating this cycle for 20, 50, and 100 times.

### 2.2. Push-Out Test and Interfacial Shear Strength

The interfacial shear strength $\bar{\tau }$ was determined from push-out tests using:

(1) $$\bar{\tau }=\frac{P_{max}}{2\pi rt}$$

where *P*_max_ is the peak push-out load, *r* is the fiber radius, and *t* is the specimen thickness. For each specimen/condition, at least five fibers were tested by the push-out method, and the interfacial shear strength was calculated from the average of the measured results to ensure the reliability of the obtained data; the corresponding scatter is shown as error bars (±SD) in the interfacial shear strength plots.

In this study, thin slices with *t* ≈ 0.5 mm were used because this thickness is commonly adopted in push-out microindentation (Bruker, Billerica, MA, USA) to enable stable fiber push-out at achievable load levels, while minimizing out-of-plane bending/buckling effects of the slice during loading. Thus, the measured response is dominated by local fiber/interphase/matrix debonding and sliding behavior around the tested fiber segment. We note that push-out results characterize local interface-sensitive behavior and may not directly represent bulk-component failure, which is additionally affected by fiber population statistics, architecture, defects/notch sensitivity, and macroscopic stress state. Accordingly, the push-out-derived interfacial shear strength is interpreted here as a local interface-sensitive indicator for comparative condition ranking, rather than a direct substitute for bulk component strength [8].

### 2.3. Microstructure Characterization

Microstructure and phase composition of the SiC_f_/AlFe5Si2 composites were examined by scanning electron microscopy (SEM, FEI, Hillsboro, OR, USA), transmission electron microscopy (TEM, JEOL Ltd., Tokyo, Japan) with energy-dispersive X-ray spectroscopy (EDS), and X-ray diffraction (XRD, Bruker AXS, Karlsruhe, Germany). Matrix Vickers hardness was measured to correlate with matrix strength. Specimens for push-out and for microstructural observation were taken from the same treatment groups.

### 2.4. Three-Dimensional FE Modeling of Thermal Residual Stresses

**Figure 1:**
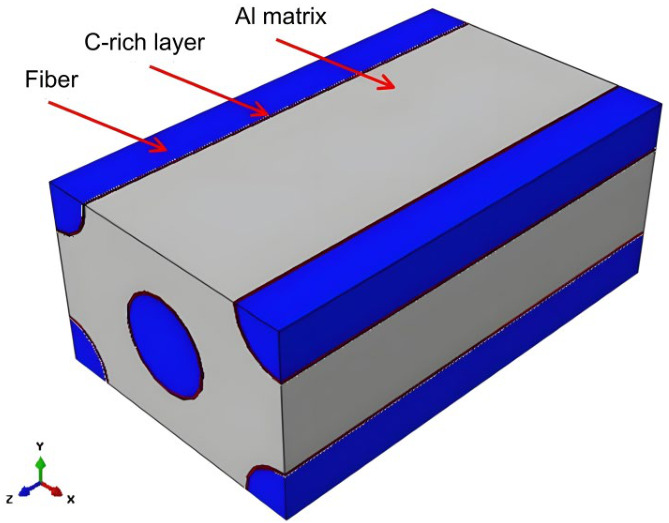
Overall 3D representative volume element (RVE) geometry used in the FE simulations, illustrating the SiC fiber, Al matrix, and C-rich layer.

Three-dimensional (3D) FE simulations (Abaqus 2024, Dassault Systèmes, Paris, France) were performed to evaluate thermal mismatch-driven residual stresses during cooling from 300, 350, 400, and 450 °C to 30 °C at a cooling rate of 10 °C/min. The 3D representative volume element (RVE) explicitly includes the SiC fiber, the Al matrix, and the C-rich layer. The model thickness along the fiber axis was set to 0.5 mm, consistent with the thickness of the push-out specimen slices. Figure 1 shows the overall 3D RVE geometry and coordinate system. For clarity of presentation, stress contours and radial path results shown in later figures are sectional/path extractions from the 3D solution.

In this work, the FE analysis was intentionally focused on the cooling stage to quantify the retained residual-stress state after thermal excursion, as a first-order mechanical indicator of interfacial stress contrast caused by thermal expansion mismatch. The contact pressure (“Pressure”) at the relevant interfaces was extracted to characterize local compressive/tensile residual stress states. It should be noted that heating-stage and dwell-stage time-dependent effects (e.g., stress relaxation, diffusion/reaction/oxidation, and possible inelastic accommodation) were not explicitly modeled in the present FE framework.

Thermo-physical properties used for the SiC fiber, aluminum alloy matrix, and C-rich layer are listed in Table 1. In the FE implementation, temperature-dependent properties were defined where tabulated data were available and were introduced using piecewise linear interpolation between adjacent temperature points. For properties without sufficient temperature-resolved data over the studied range, constant values were used. This discretized property assignment may introduce interpolation uncertainty, particularly when a property exhibits strong nonlinearity with temperature. Therefore, the FE results are interpreted primarily as semi-quantitative trend indicators (e.g., variation in residual-stress magnitude with peak temperature), rather than fully calibrated absolute residual-stress values. Recent advances in finite element modeling of residual stresses in fiber-reinforced composites provide a broader methodological context for the present analysis [9,10].

## 3. Results and Discussion

### 3.1. Effect of Thermal Exposure on Interfacial Shear Strength

**Figure 2:**
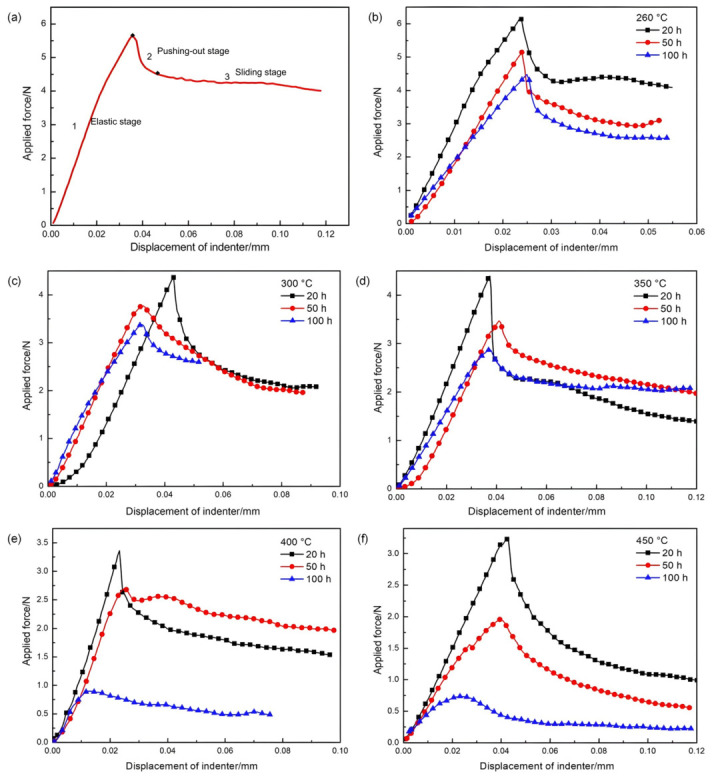
Applied load versus displacement curves of the SiC_f_/AlFe5Si2 composite specimens (0.5 mm thick): (**a**) without thermal exposure; (**b**) with exposure at 260 °C for 20/50/100 h; (**c**) with exposure at 300 °C for 20/50/100 h; (**d**) with exposure at 350 °C for 20/50/100 h; (**e**) with exposure at 400 °C for 20/50/100 h; (**f**) with exposure at 450 °C for 20/50/100 h.

Figure 2 shows representative load–displacement curves from push-out tests on SiC_f_/AlFe5Si2 specimens (0.5 mm thick) in different thermal-exposure conditions. As shown in Figure 2a, the push-out process could be divided into three stages: (i) a linear load–displacement increase associated with interfacial deformation; (ii) a rapid load drop corresponding to fiber debonding/push-out; and (iii) a gradual load decrease dominated by interfacial sliding friction. Using Equation (1), the interfacial shear strength of the as-received specimen was ~32 MPa. Figure 2b–f present curves after thermal exposure at 260, 300, 350, 400, 450 °C for 20, 50, 100 h, showing a clear reduction in peak load with increasing temperature and time.

As summarized in our previous work [7], thermal exposure reduced the interfacial shear strength, and the reduction became more pronounced as either temperature or time increased. Below 350 °C, the reduction remained moderate (within ~35%). When the exposure temperature exceeded 400 °C, the interfacial shear strength decreased sharply, as reflected by the increased slope of the strength-time curves. After exposure at 400 °C for 50 h, the interfacial shear strength dropped by ~50% compared with the as-received condition. These results suggest that the SiC_f_/AlFe5Si2 composite can potentially be used at temperatures up to 350 °C, which is significantly higher than the typical service limit of conventional aluminum alloys (~200 °C). This finding is consistent with recent studies on thermal cycling behavior of SiC/Al composites [16].

**Figure 3:**
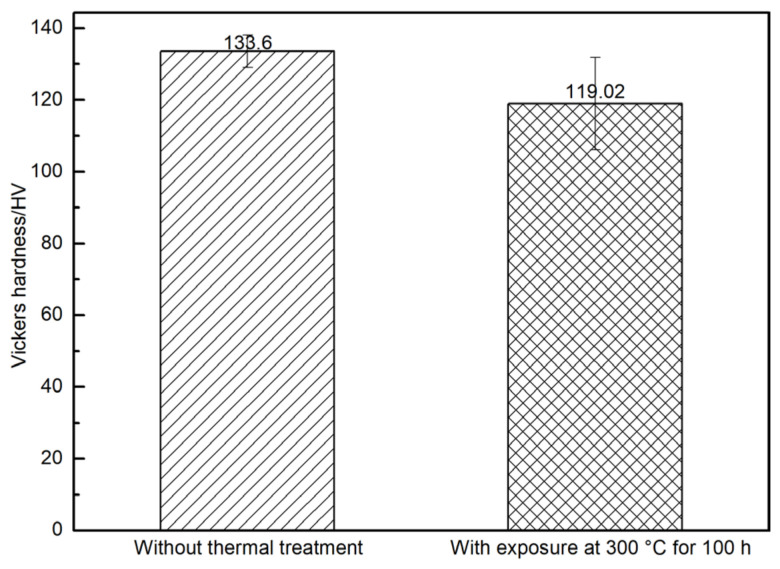
Vickers hardness of the matrix alloy in SiC_f_/AlFe5Si2 composites without thermal treatment and with exposure at 300 °C for 100 h.

To relate the interfacial strength degradation to matrix softening, Figure 3 compares the Vickers hardness of the matrix alloy before and after exposure at 300 °C for 100 h. The average hardness decreased from 133.6 HV to 119.0 HV. Since tensile strength correlates with hardness σ ≈ 3HV ± 5% HV [17], matrix strength degradation during thermal exposure likely reduced the radial constraint and contact stress around fibers (via Poisson effects during push-out), thereby decreasing the apparent interfacial shear strength. This interpretation is consistent with the observation that the variation in interfacial shear strength follows the same trend as the tensile strength of the Al matrix [8].

### 3.2. Microstructural Evolution During Thermal Exposure

**Figure 4:**
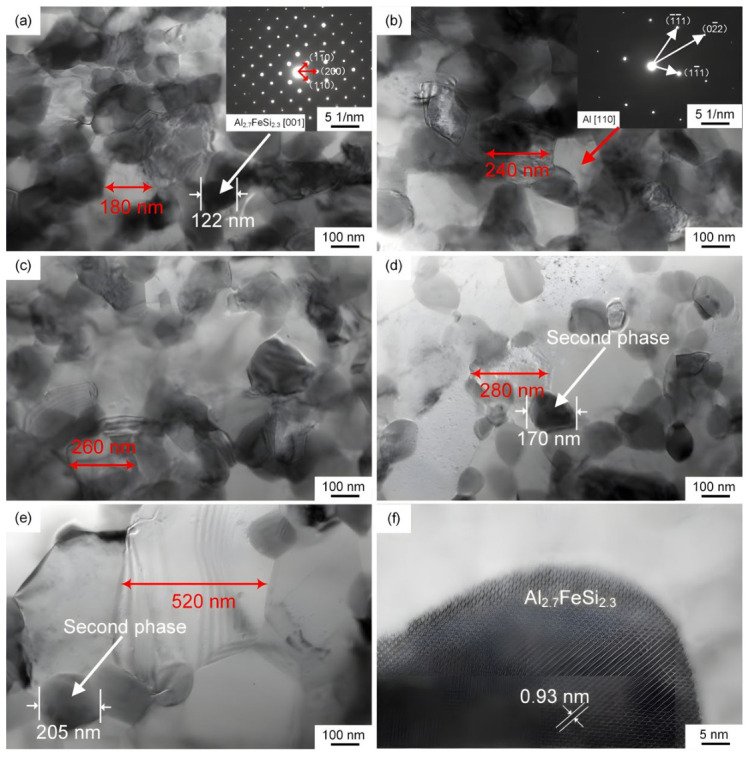
TEM images of the AlFe5Si2 matrices in the SiC_f_/AlFe5Si2 composite (**a**) without exposure; (**b**) with exposure at 300 °C for 20 h; (**c**) with exposure at 300 °C for 50 h; (**d**) with exposure at 450 °C for 20 h; (**e**) with exposure at 450 °C for 50 h; (**f**) lattice spacing of the second phase.

Figure 4 shows TEM images of the AlFe5Si2 matrices in the SiC_f_/AlFe5Si2 composite before and after exposure at 300 and 450 °C for 20 and 50 h, respectively. It can be observed that the grain size of the aluminum alloy matrix in the composite without thermal exposure was approximately 180 nm (see Figure 4a), which grew to 240 nm and 260 nm after thermal exposure at 300 °C for 20 h and 50 h, respectively, and grew to 280 nm and 520 nm after thermal exposure at 450 °C for 20 h and 50 h, respectively. The experimental results indicate that grain size increases with increasing thermal exposure temperature and time. Additionally, Al_2.7_FeSi_2.3_ phases existed at the grain boundaries of the composite matrix aluminum alloy without thermal exposure, and with increasing thermal exposure temperature and time, the second phase size slightly increased, but the quantity decreased. Vickers hardness tests showed that thermal exposure reduced the strength of the composite matrix.

**Figure 5:**
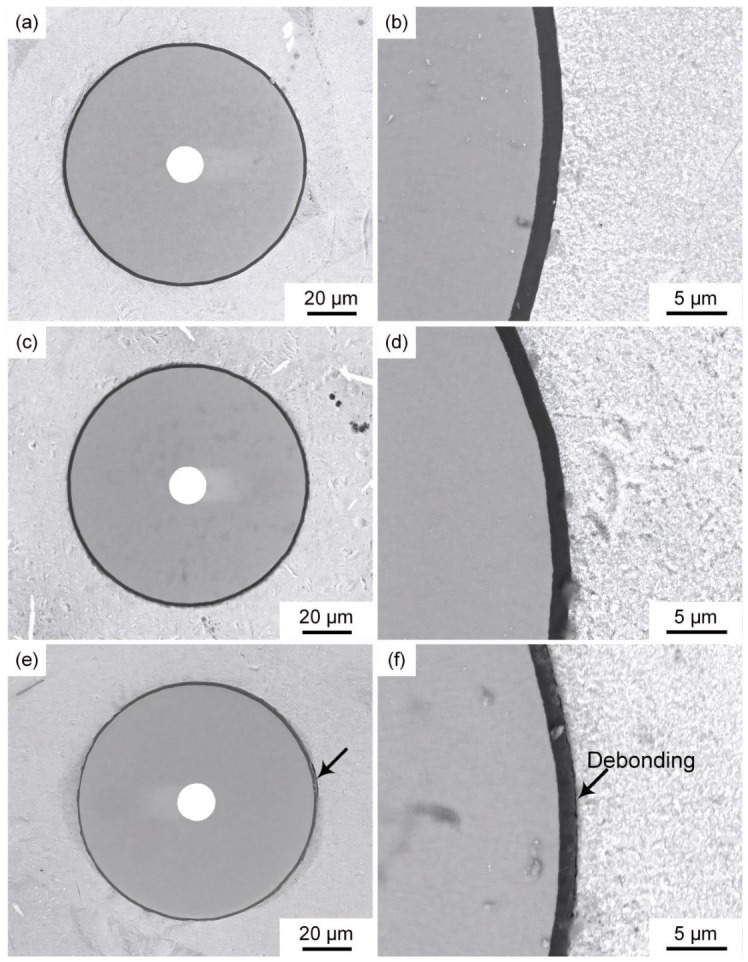
Microstructures of the SiC_f_/AlFe5Si2 composites thermally exposed at: (**a**) 300 °C; (**c**) 350 °C; (**e**) 400 °C for 50 h; and (**b**,**d**,**f**) their magnification, respectively. The arrows indicate the interface debonding observed in the microstructures.

Figure 5 shows microstructures of SiC_f_/AlFe5Si2 composites after exposure at 300, 350, and 400 °C for 50 h. No interfacial cracks or apparent defects were observed after exposure at 300 and 350 °C (Figure 5b,d), consistent with the relatively small reduction in interfacial shear strength. In contrast, interfacial debonding occurred after exposure at 400 °C for 50 h (Figure 5e,f), which corresponded to a ~50% decrease in interfacial shear strength.

**Figure 6:**
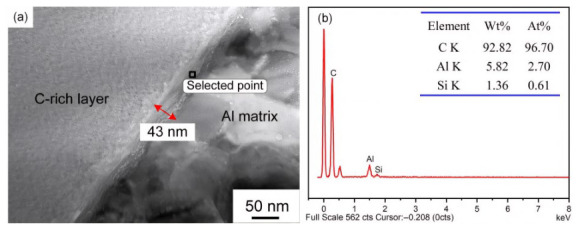
SiC_f_/AlFe5Si2 composites without exposure: (**a**) interface microstructure; (**b**) EDS analysis of the selected point in the interface zone.

**Figure 7:**
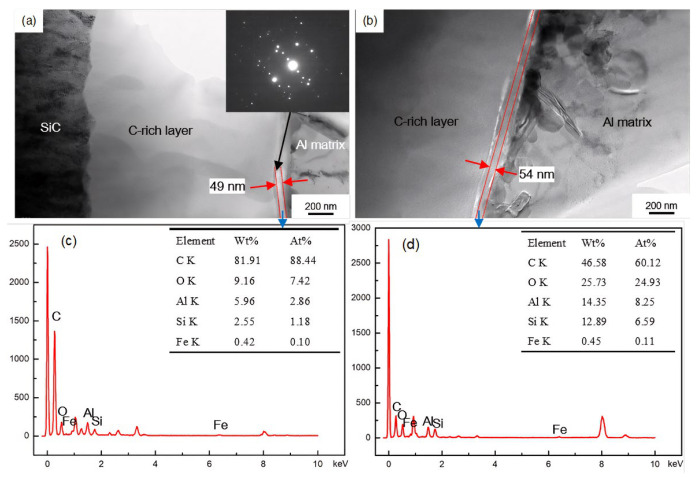
TEM images of interface microstructures of the SiC_f_/AlFe5Si2 composites exposed at (**a**) 300 °C for 20 h; (**b**) 300 °C for 100 h; (**c**,**d**) EDS analyses of the selected areas in the interface zone, respectively.

**Figure 8:**
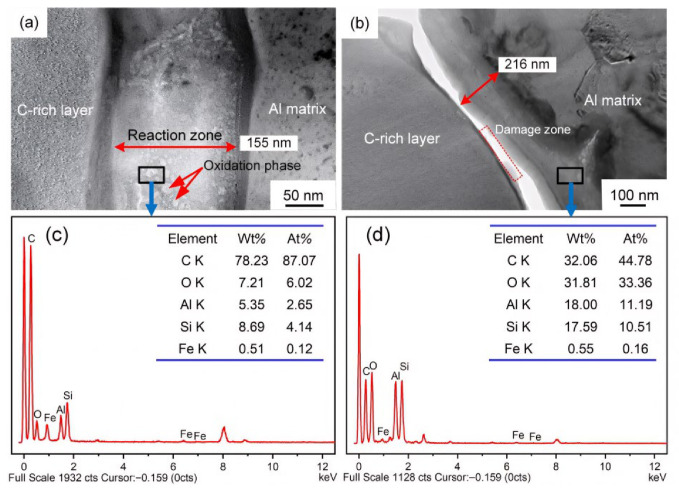
TEM images of interface microstructures of the SiC_f_/AlFe5Si2 composites exposed at (**a**) 450 °C for 20 h; (**b**) 450 °C for 50 h; (**c**,**d**) EDS analyses of the selected areas in the interface zone, respectively [7].

TEM/EDS analyses (Figure 6, Figure 7 and Figure 8) revealed that the interfacial reaction layer between the C-rich layer and the Al matrix was ~43 nm thick in the as-received state. After exposure at 300 °C for 20 and 100 h, the thickness increased slightly to ~49 and ~54 nm, respectively, and diffraction patterns indicated that the reaction product was mainly Al_4_C_3_. In contrast, exposure at 450 °C for 20 and 50 h led to substantial growth of the reaction layer to ~155 and ~216 nm, respectively. Previous studies have shown that, above 400 °C, brittle Al_x_SiO_y_ oxides can form at the interface and severely degrade interfacial shear strength and overall performance [18,19], which is consistent with our earlier XRD results on SiC_f_/AlFe5Si2 composites [7]: after exposure at 260–350 °C for 50 h, the main phases were Al, SiC, W, C, and Al_4_C_3_, whereas additional Al_x_SiO_y_ phases (e.g., Al_3.21_SiO_y_) appeared after exposure above 400 °C for 50 h. It should be noted that the propensity for such oxide formation is influenced by oxygen availability; therefore, oxide-related degradation may be mitigated under inert/vacuum conditions or with effective coatings. The damaged zone adjacent to the reaction layer (Figure 8b was therefore likely associated with interfacial debonding induced by the thickened reaction layer and the presence of brittle oxides.

**Figure 9:**
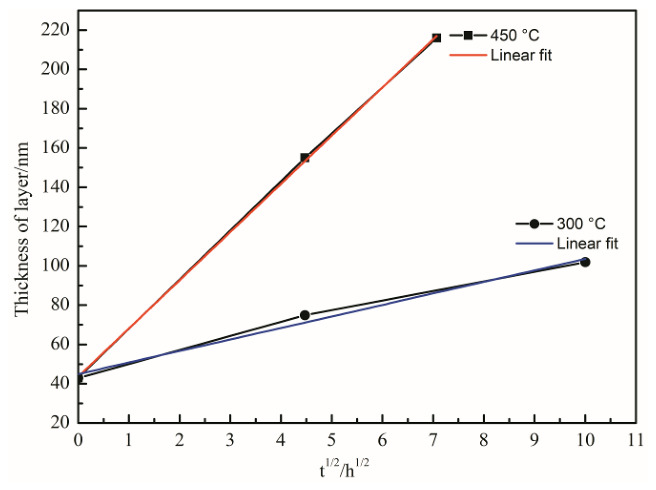
Interfacial reaction kinetic curves of the SiC_f_/AlFe5Si2 composite exposed at 300 and 450 °C.

Interfacial reactions were reaction-controlled at early stages and became diffusion-controlled after a thin reaction layer formed [19]. The layer growth followed a parabolic law [20,21]:

*l* = (*kt*)^1/2^ + *b*_0_ (2)

where *l* is the reaction-layer thickness, *k* is the growth-rate constant, *t* is exposure time, and *b*_0_ is the initial thickness. Linear fits of *l* versus *t*^1/2^ (Figure 9) yielded:

*l*_1_ *=* 0.95*t*^1/2^ *+* 43 (3)

*l*_2_ *=* 24.5*t*^1/2^ *+* 43 (4)

with *l* in nm and *t* in h, resulting in *k*_1_ = 3.249 × 10^−15^ m^2^/s at 300 °C and *k*_2_ = 2.161 × 10^−12^ m^2^/s at 450 °C. The temperature dependence of *k* was described by the Arrhenius relationship [2]:

*k* = *A*exp(−*Q*/*RT*) (5)

where *A* is the pre-exponential factor, *Q* is the activation energy, *R* is the gas constant, and *T* is the absolute temperature. Using *T*_1_ = 573 K and *T*_2_ = 723 K, the activation energy was estimated to be *Q* ≈ 150 kJ/mol. It should be noted that this Arrhenius evaluation is based on only two temperatures (300 °C and 450 °C). These two temperatures were selected because they provided clearly distinguishable low- and high-temperature interfacial growth behaviors with measurable reaction-layer thickness changes under the current TEM-based quantification protocol. Therefore, the obtained *Q* represents a preliminary apparent activation energy and should be interpreted as an order-of-magnitude indicator rather than a uniquely constrained kinetic parameter; additional isothermal temperatures would be required to improve statistical reliability and to assess potential mechanism changes. Despite the above limitations, the apparent *Q* is broadly within the range reported in related SiC/Al interfacial reaction studies (e.g., 132 kJ/mol in [22] and 180–190 kJ/mol in [2,23]), providing a qualitative consistency check for the present kinetic estimate. The observed kinetic behavior is consistent with recent studies on interfacial reaction kinetics in SiC fiber reinforced aluminum composites [17].

Based on the above analysis, it can be concluded that the interfacial bonding performance of SiC_f_/AlFe5Si2 composite materials is related to the thermodynamic and kinetic changes in the matrix and interfacial reaction zones. As the thermal exposure temperature and time increase, the interfacial reaction zone in the composite materials becomes increasingly thick. This is especially evident when the thermal exposure temperature exceeds 400 °C, the Al_x_SiO_y_ phase is formed in the interfacial reaction zone, and this brittle phase will severely weaken the interfacial bonding force. The thermal exposure process causes the composite matrix grain size to increase and strength to decrease, resulting in reduced interfacial shear strength. The thermal exposure test results show that the service temperature of SiC_f_/AlFe5Si2 composite materials should not exceed 350 °C.

It should be emphasized that strict quantitative decoupling of degradation contributions is beyond the scope of the present dataset, because matrix softening, interfacial reactions, and brittle phase formation evolve concurrently. Therefore, a semi-quantitative attribution framework is adopted here by integrating multiple evidence streams: matrix hardness reduction (softening indicator), TEM-observed reaction-layer thickening and interfacial phase evolution (reaction indicator), XRD/EDS identification of Al_x_SiO_y_ (brittle-phase indicator), and push-out interfacial shear strength (integrated response metric). From this combined evidence, the dominant mechanisms differ by temperature regime: below ~350 °C, degradation is moderate and mainly associated with matrix softening plus mild interfacial reaction; above ~400 °C, sharp degradation is consistent with rapid reaction-layer growth, brittle Al_x_SiO_y_ formation, and interfacial debonding damage. To clarify the relative roles of matrix softening, interfacial reaction-layer growth, and brittle oxide formation in the observed interfacial degradation, a mechanism-oriented summary is provided in Table 2.

### 3.3. Effect of Thermal Cycling and Residual Stresses

**Figure 10:**
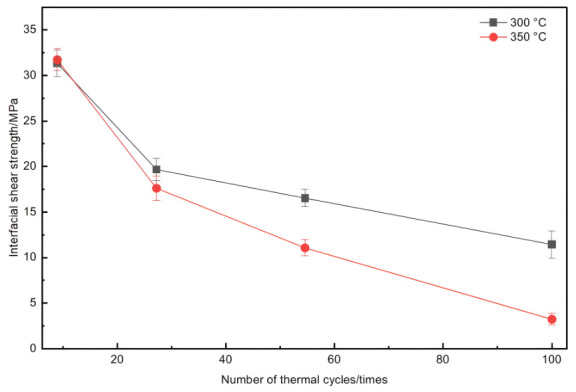
Interfacial shear strength versus number of thermal cycles curve of the SiC_f_/AlFe5Si2 composites without thermal exposure and thermal cycled at 300/350 °C for 20/50/100 times.

As summarized in Table 2, the dominant degradation drivers shift from matrix softening/mild interfacial reaction at ≤350 °C to brittle-phase-assisted interfacial damage at ≥400 °C. Figure 10 compares interfacial shear strength after thermal cycling at 300 and 350 °C. Thermal cycling led to more severe degradation than thermal exposure at the same temperature and comparable total time. For example, at 300 °C, 20/50/100 cycles reduced the strength from 32 MPa to 25/23/20 Mpa (reductions of 22%/28%/37%), whereas thermal exposure for 20/50/100 h reduced it to 27/26/23 Mpa (18%/21%/28%). At 350 °C, 100 cycles reduced the strength by ~53%, indicating that the interface became too weak for reliable service. These results suggest that the recommended service temperature under thermal cycling should not exceed 300 °C.

**Figure 11:**
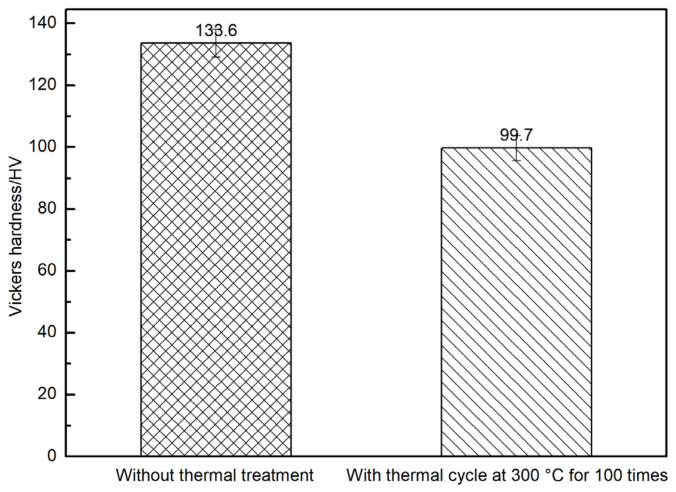
Vickers hardness of the matrix alloy in SiC_f_/AlFe5Si2 composites without thermal treatment and with thermal cycle at 300 °C for 100 times.

Hardness measurements (Figure 11) showed that after 100 cycles at 300 °C, the matrix hardness decreased from 133.6 HV to 99.7 HV, which was a larger reduction than that caused by 100 h exposure at 300 °C. Combined with [8,17], this supports that cyclic thermal loading accelerates matrix softening and interfacial degradation.

**Figure 12:**
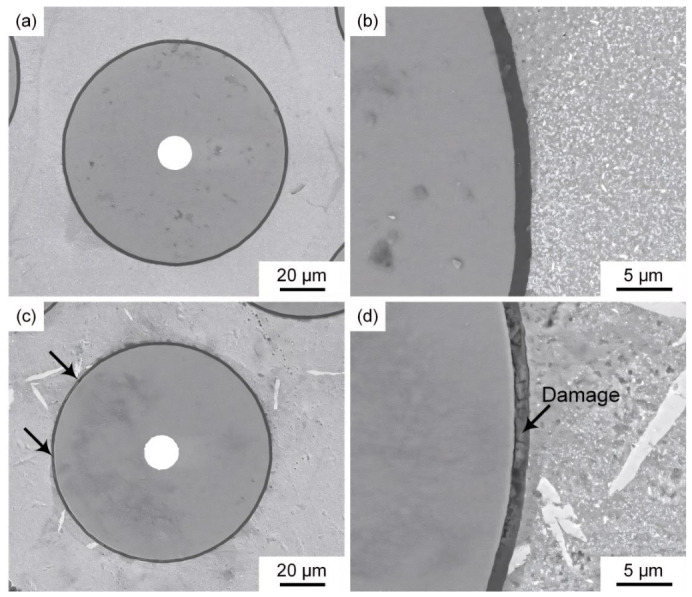
Microstructures of the SiC_f_/AlFe5Si2 composites thermally cycled at: (**a**) 300 °C for 100 times; (**c**) 350 °C for 100 times; and (**b**,**d**) their magnification, respectively. The arrows indicate the interface damage observed in the microstructures.

Figure 12 shows microstructures of the SiC_f_/AlFe5Si2 composites thermally cycled at 300 and 350 °C for 100 times. It can be observed that the C/Al interface of the SiC_f_/AlFe5Si2 composite thermally cycled at 300 °C for 100 times showed no cracks or other defects (see Figure 12a,b), and the carbon layer was undamaged. The corresponding interfacial shear strength of the composite did not decrease significantly (~34%). The carbon layer of the SiC_f_/AlFe5Si2 composite thermally cycled at 350 °C for 100 times was ruptured and severely damaged (see Figure 12c,d), and the corresponding interfacial shear strength of the composite was reduced by about 53% compared with that of the composite without thermal cycling.

**Figure 13:**
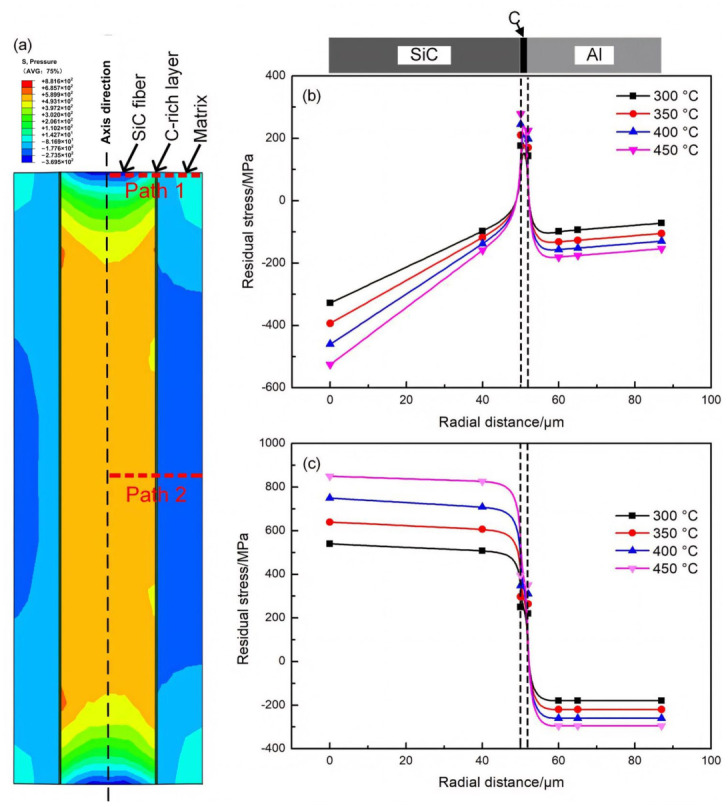
Residual stress (pressure/contact pressure) results obtained from the 3D FE model and presented as a representative sectional contour and extracted radial path profiles: (**a**) stress contour on a representative plane; (**b**) stress distribution along path 1; (**c**) stress distribution along path 2.

Interfacial bonding in MMCs is closely related to residual stresses [24,25]. In this work, the 3D FEM analysis is intentionally cooling-focused: cooling from 300/350/400/450 °C to 30 °C at 10 °C/min was simulated to evaluate mismatch-driven retained residual stresses (Figure 13; shown as representative sectional/path extractions from the 3D solution). This cooling-focused FE treatment provides a first-order mechanical indicator of interfacial stress contrast. However, thermal-cycling degradation is governed by the full thermal history; heating and high-temperature dwell can introduce additional stress relaxation and time-dependent effects (e.g., diffusion/reaction/oxidation and possible creep/plastic accommodation), which were not explicitly included in the current FE model. Therefore, the FE results are interpreted as a mechanistic complement to experiments rather than a standalone full-life prediction of cycling damage.

The results showed that the matrix experienced radial tensile stresses, whereas the C-rich layer experienced radial compressive stresses. Increasing the peak temperature increased the magnitude of residual stresses. Large opposing stresses between the matrix and C-rich layer could promote debonding at the interface during cooling, consistent with the debonding observed after 400 °C exposure (Figure 5e,f). During thermal cycling, alternating stress states repeatedly acted on the matrix and C-rich layer, aggravating debonding. Moreover, the C-rich layer (~2 μm thick) exhibited a large stress gradient, making it prone to crack initiation under cyclic loading, which was consistent with the severe carbon-layer damage after 100 cycles at 350 °C (Figure 12c,d). Together with grain growth/coarsening-induced matrix softening and interfacial oxidation producing brittle Al_x_SiO_y_ phases, these factors collectively led to the pronounced reduction in interfacial shear strength. The residual stress analysis methodology employed here follows recent advances in finite element modeling of fiber-reinforced composites [10] and is also consistent with recent SiC/Al studies on residual-stress evolution under thermal-process histories [26]. These observations are consistent with the semi-quantitative mechanism attribution proposed in Section 3.2, where high-temperature (>~400 °C) degradation is dominated by coupled brittle-phase-assisted interfacial damage and accelerated reaction-layer thickening.

The proposed temperature limits in this study are derived from combined experimental indicators rather than from a single criterion. Specifically, we considered: (i) retention of push-out interfacial shear strength relative to the as-received condition, (ii) interfacial microstructural integrity (debonding and C-rich layer damage), (iii) the relative severity of thermal cycling versus isothermal exposure, and (iv) onset of sharp degradation associated with accelerated reaction-layer thickening and brittle-phase-related damage. Under these criteria, ~350 °C is identified as a practical upper guidance level for isothermal exposure in the present test framework, while ~300 °C is recommended under thermal cycling due to stronger cumulative degradation.

### 3.4. Limitations and Applicability of the Simulated Service Environments

The thermal exposure and thermal cycling protocols adopted in this work were designed to reproduce, in a controlled and repeatable manner, the dominant thermal histories relevant to the low-temperature section of aero-engine fan/compressor components, while enabling systematic quantification of interfacial shear strength degradation and microstructural evolution. We acknowledge that actual aerospace service environments can involve coupled thermo-mechanical loading (e.g., vibration/fatigue, foreign object impact, and stress-assisted creep), complex atmospheres, and additional degradation mechanisms (e.g., erosion, moisture-, salt-, or contaminant-assisted corrosion), which were not explicitly included here. Therefore, the present study intentionally isolates thermal variables (peak temperature, dwell time, and number of cycles) to establish baseline trends for (i) matrix softening, (ii) interfacial reaction-layer growth, and (iii) oxidation-related brittle phase formation at elevated temperatures.

All thermal treatments were conducted in air, which provides a practical upper-bound or conservative condition for oxidation-driven interfacial degradation in Al-based composites. Because oxygen availability in air can accelerate oxide formation at the interface, the extent of Al_x_SiO_y_-related embrittlement observed here should be considered environment-dependent. Consequently, the formation of brittle Al_x_SiO_y_ phases and the associated interfacial strength loss observed above ~400 °C may be less pronounced under inert/vacuum conditions or when effective environmental barrier coatings are applied. Nonetheless, the relative ranking between isothermal exposure and thermal cycling, as well as the mechanistic roles of thermal mismatch stresses, matrix softening, and interphase damage, remain informative in defining safe thermal operating windows and for guiding interfacial stabilization strategies. This interpretation is consistent with broader thermal-cycling studies in light-alloy composites, where cumulative thermo-mechanical history governs crack initiation and damage progression [27]. Future work will consider combined thermo-mechanical cycling and alternative atmospheres to further bridge laboratory simulations and in-service conditions.

Regarding generalizability, the present conclusions are most directly applicable to the SiC_f_/AlFe5Si2 composite with a C-rich layer prepared by a similar processing route and evaluated under air thermal exposure/cycling conditions. Extrapolation to other Al-based MMC systems should be made with caution, because different matrix chemistries may show different oxidation susceptibility, interfacial reaction pathways/kinetics, and high-temperature matrix softening behavior. Therefore, the proposed service limits (350 °C under isothermal exposure and 300 °C under thermal cycling) are intended as material-specific engineering indicators for the present system, rather than universal thresholds for all SiC_f_/Al composites. Qualitatively, the observed trend—more severe degradation under thermal cycling than under isothermal exposure at comparable temperatures—is consistent with prior MMC studies, but quantitative cross-system comparison requires dedicated side-by-side experiments.

These temperature limits should be interpreted as conservative engineering indicators under the present experimental boundary conditions (air environment, no externally applied mechanical load, and push-out-based local interfacial assessment). Because real components experience coupled thermo-mechanical and environmental effects, the proposed limits are not universal constants and should be refined through application-specific validation.

## 4. Conclusions

This chapter simulated the service working environment of SiC_f_/AlFe5Si2 composite materials through thermal exposure and thermal cycling experiments. The effects of simulated service environments on the microstructure and interfacial properties of the composite materials were studied. The following conclusions were obtained:

(1) Thermal exposure degraded the SiC_f_/AlFe5Si2 interface, with a marked acceleration above ~400 °C. This degradation is associated with reaction-layer thickening, brittle Al_x_SiO_y_ formation, and matrix softening. The reaction layer followed parabolic growth, and a preliminary two-temperature Arrhenius estimate gave an apparent activation energy of ~150 kJ/mol.
(2) Thermal cycling caused stronger interfacial deterioration than isothermal exposure at comparable temperature/time levels. Based on interfacial shear-strength retention and observed microstructural integrity, 350 °C (exposure) and 300 °C (cycling) are proposed as conservative, condition-specific engineering limits for the present material system.
(3) Cooling-focused 3D FEM indicated increasing matrix/C-rich-layer stress contrast with increasing peak temperature, supporting a mechanical driving force for interfacial debonding. Since heating/dwell time-dependent effects were not explicitly modeled, the FE results are interpreted as mechanistic trend support rather than standalone life prediction.

## Figures and Tables

**Table 1:** Thermo-physical properties of SiC fiber, aluminum alloy matrix and C-rich layer.

Materials	Thermal Conductivity (W/mK)	Specific Heat Capacity (SHC) (J/(kg·°C))		Coefficient of Thermal Expansion (CTE) (10^−6^·°C^−1^)	
		Temperature (°C)	SHC	Temperature (°C)	CTE
SiC [11,12]	83.6	107	472	2.6	
		247	1267		
aluminum alloy [12,13]	203	880		100	22.7
				200	23.8
				300	24.8
				400	25.9
C-rich layer [14,15]	129	710		3	

Notes: (a) Temperature-dependent properties were implemented in the FE model by piecewise linear interpolation between adjacent tabulated temperature points. (b) For properties lacking sufficient temperature-resolved datasets within the studied range, constant values were used as listed. (c) No extrapolation beyond the tabulated temperature interval was applied in this work. (d) Due to discretized property input, interpolation-related uncertainty may affect local stress magnitudes; therefore, FE outputs are interpreted primarily as semi-quantitative trend indicators rather than fully calibrated absolute residual-stress values.

**Table 2:** Mechanism-oriented attribution of interfacial degradation in SiC_f_/AlFe5Si2 under thermal exposure/cycling.

Condition/Temperature Regime	Dominant Evidence	Primary Degradation Driver(s)	Net Effect on Interfacial Shear Strength
≤350 °C (thermal exposure)	Matrix hardness drop + slight reaction-layer thickening (TEM)	Matrix softening + mild interfacial reaction	Moderate reduction
>400 °C (thermal exposure)	Rapid layer thickening (TEM) + Al_x_SiO_y_ emergence (XRD/EDS) + debonding morphology	Brittle oxide formation + accelerated interfacial reaction	Sharp reduction
Thermal cycling at 300 °C	Larger hardness loss than isothermal + cyclic stress alternation (FEM-supported trend)	Cumulative softening + cyclic stress-assisted damage	Greater reduction than isothermal at the same T
Thermal cycling at 350 °C	Carbon-layer rupture/cracking + high stress-gradient indication	Interphase cracking + progressive debonding under cyclic loading	Most severe reduction

## Data Availability

The original contributions presented in this study are included in the article. Further inquiries can be directed to the corresponding author.

## References

[1] Wang H., Xing W., Chen S., Song C., Dickey M.D., Deng T. (2021). Liquid metal composites with enhanced thermal conductivity and stability using molecular thermal linker. Adv. Mater..

[2] Liu H., Madaleno U., Shinoda T., Mishima Y., Suzuki T. (1990). Interfacial reaction and strength of SiC fibres coated with aluminium alloys. J. Mater. Sci..

[3] Zhang Y., Hu J., Dong S., Li Y. (2024). Influence of the thickness of pyrolytic carbon interphase on the mechanical behavior of SiC/(BN/PyC)/SiC composites. Ceram. Int..

[4] Zhong Z., Jiang X., Sun H., Wu Z., Yang L., Matamoros-Veloza A. (2024). Recent research on the optimization of interfacial structure and interfacial interaction mechanisms of metal matrix composites: A review. Mater. Adv. Eng. Mater..

[5] Chen L.G., Lin S.J., Chang S.Y. (2005). Analysis of interfacial shear strength of SiC fiber reinforced 7075 aluminum composite by pushout microindentation. Metall. Mater. Trans. A.

[6] Jiang Y.Q., Liu J.H., Wu X.B. (1994). A measure of interfacial frictional shear strength of MMC. Chin. J. Mater. Res..

[7] Chu D.S., Ma Y., Tang P.J., Li P.Y. (2020). Effect of thermal exposure on the interface microstructure and interfacial shear strength of the SiC fiber reinforced AlFe5Si2 matrix composite. Appl. Compos. Mater..

[8] Kozera R., Boczkowska A., Krawczyk Z.D., Kozera P., Spychalski M., Malek M., Kosturek R. (2021). Push-Out Method for Micro Measurements of Interfacial Strength in Aluminium Alloy Matrix Composites. Materials.

[9] Wang Q., Zhu Z., Maryum P., Liu Y., Xue T., Lu C., Hu B., Zhang C., Qin R. (2021). Finite element analysis of the influence of interphase on the thermal residual stress level and distribution of SiCp/6061Al composites. Compos. Interfaces.

[10] Jiang Z., Lian J., Yang D., Dong S. (1998). An analytical study of the influence of thermal residual stresses on the elastic and yield behaviors of short fiber-reinforced metal matrix composites. Mater. Sci. Eng..

[11] Elkind A., Barsoum M.W., Kangutkar P. (1992). Thermal expansion of silicon carbide monofilaments and silicon carbide–borosilicate composites. J. Am. Ceram. Soc..

[12] Chen J.K., Hung H.Y., Wang C.F., Tang N.K. (2015). Thermal and electrical conductivity in Al–Si/Cu/Fe/Mg binary and ternary Al alloys. J. Mater. Sci..

[13] Mo L., Lin M., Zhan M., Zhao Y.-J., Du J. (2024). Experimental and theoretical study of the microstructure evolution and thermal-physical properties of hypereutectic Al–Fe alloys. J. Mater. Res..

[14] Null M.R., Lozier W.W., Moore A.W. (1973). Thermal diffusivity and thermal conductivity of pyrolytic graphite from 300 to 2700 K. Carbon.

[15] Touloukian Y.S., Kirby R.K., Taylor E.E., Desai P.D. (1977). Thermophysical Properties of Matter.

[16] Lin X., Xu Q., Deng T., Yang B., Chen L. (2024). Improving thermal conductivity of Al/SiC composites by post-oxidization of reaction-bonded silicon carbide preforms. Sci. Rep..

[17] Yasutomi Y., Sawada J., Kikuchi T., Nakamura K., Manabe Y., Nagano K., Kuroda H., Sumi T., Kubokawa H., Nagai M. (1999). Effects of the SiC/Al interface reaction on fracture behavior of a composite conductor using SiC fiber reinforced aluminum for next generation power equipment. J. Mater. Sci..

[18] Ochiai S., Osamura K. (1989). Tensile strength of fibre-reinforced metal matrix composites with non-uniform fibre spacing. J. Mater. Sci..

[19] Guo Z.X., Derby B. (1994). Interfaces in Ti3Al composites reinforced with Sigma SiC fibers. Scr. Metall. Mater..

[20] Lü X.-H., Yang Y.-Q., Ma Z.-J., Liu C.-X., Chen Y., Ai Y.-L. (2006). Kinetics and mechanism of interfacial reaction in SCS-6 SiC continuous fiber-reinforced Ti-Al intermetallic matrix composites. Trans. Nonferrous Met. Soc. China.

[21] Breuer J., Wilger T., Friesel M., Herzig C. (1999). Interstitial and substitutional diffusion of metallic solutes in Ti3Al. Intermetallics.

[22] Yasutomi Y., Sawada J., Iwai K., Hase Y., Nagano K., Kuroda H., Sumi T., Kogure H., Sawai Y., Kishi T. (1998). Mechanical properties of SiC fiber after immersion in molten aluminum. J. Ceram. Soc. Jpn..

[23] Liu J.A. (2012). Conversion and substitution of various mechanical property indexes of aluminum alloy materials. Light Alloy Fabr. Technol..

[24] Ananth C.R., Chandra N. (1995). Evaluation of interfacial shear properties of metal matrix composites from fibre push-out tests. Mech. Adv. Mater. Struct..

[25] Chandra N., Ananth C.R. (1995). Analysis of interfacial behavior in MMCs and IMCs by the use of thin-slice push-out tests. Compos. Sci. Technol..

[26] Li B., Ouyang W., Dong Y. (2024). Investigating the Effects and Mechanisms of Thermal–Vibration-Coupled Stress Relief Treatment on Residual Stress in SiC/Al Composites. Metals.

[27] Mousavi S.F., Sharifi H., Tayebi M., Hamawandi B., Behnamian Y. (2022). Thermal cycles behavior and microstructure of AZ31/SiC composite prepared by stir casting. Sci. Rep..

